# Role of Impaired Glycolysis in Perturbations of Amino Acid Metabolism in Diabetes Mellitus

**DOI:** 10.3390/ijms24021724

**Published:** 2023-01-15

**Authors:** Milan Holeček

**Affiliations:** Department of Physiology, Faculty of Medicine, Charles University, 500 03 Hradec Králové, Czech Republic; holecek@lfhk.cuni.cz

**Keywords:** branched-chain amino acids, serine, glycine, insulin resistance

## Abstract

The most frequent alterations in plasma amino acid concentrations in type 1 and type 2 diabetes are decreased L-serine and increased branched-chain amino acid (BCAA; valine, leucine, and isoleucine) levels. The likely cause of L-serine deficiency is decreased synthesis of 3-phosphoglycerate, the main endogenous precursor of L-serine, due to impaired glycolysis. The BCAA levels increase due to decreased supply of pyruvate and oxaloacetate from glycolysis, enhanced supply of NADH + H^+^ from beta-oxidation, and subsequent decrease in the flux through the citric acid cycle in muscles. These alterations decrease the supply of α-ketoglutarate for BCAA transamination and the activity of branched-chain keto acid dehydrogenase, the rate-limiting enzyme in BCAA catabolism. L-serine deficiency contributes to decreased synthesis of phospholipids and increased synthesis of deoxysphinganines, which play a role in diabetic neuropathy, impaired homocysteine disposal, and glycine deficiency. Enhanced BCAA levels contribute to increased levels of aromatic amino acids (phenylalanine, tyrosine, and tryptophan), insulin resistance, and accumulation of various metabolites, whose influence on diabetes progression is not clear. It is concluded that amino acid concentrations should be monitored in patients with diabetes, and systematic investigation is needed to examine the effects of L-serine and glycine supplementation on diabetes progression when these amino acids are decreased.

## 1. Introduction

Diabetes mellitus occurs in two basic forms—diabetes of the first type (T1DM, type 1 diabetes mellitus) and diabetes of the second type (T2DM, type 2 diabetes mellitus). The cause of T1DM, which usually manifests itself in young individuals (juvenile diabetes), is insufficient insulin production in the β-cells of the islets of Langerhans. In T2DM, the effects of insulin are counteracted by factors that induce a state of insulin resistance. In the early stage, the increased output of insulin from β-cells compensates the insulin insensitivity. In later stages, a defect in insulin secretion develops, and therapy may require insulin administration. T2DM is becoming increasingly common in obese children [1].

Both types of diabetes develop marked disturbances in amino acid metabolism and amino acid concentrations in plasma and tissues. However, alterations are not consistent for most amino acids. Most consistently increase the levels of branched-chain amino acids (BCAA; valine, leucine, and isoleucine) and aromatic amino acids (AAA, phenylalanine, tyrosine, and tryptophan) and decrease the levels of L-serine and glycine [2,3,4]. There are inconsistent data on changes in alanine, glutamate, aspartate, and glutamine, although these amino acids play a role in BCAA catabolism reactions [4,5,6,7].

Although it is supposed that disturbances in aminoacidemia play a role in the development of diabetes and its complications, their pathogenesis is not completely clear. Important roles have undoubtedly alterations in protein balance, food intake, amino acid transport through cell membranes, and increased gluconeogenesis in the liver and kidneys. The aims of the present article are (1) to demonstrate that decreased glycolysis and preferential fatty acid oxidation, and subsequent decrease in the flux trough citric acid cycle (CAC) are the main causes of decreased L-serine and increased BCAA levels in diabetes and (2) examine the contribution of disturbances in L-serine and BCAA metabolism in the pathogenesis of altered concentrations of other amino acids and diabetes-associated complications.

## 2. Basic Data on Glycolysis and the CAC

**Figure 1:**
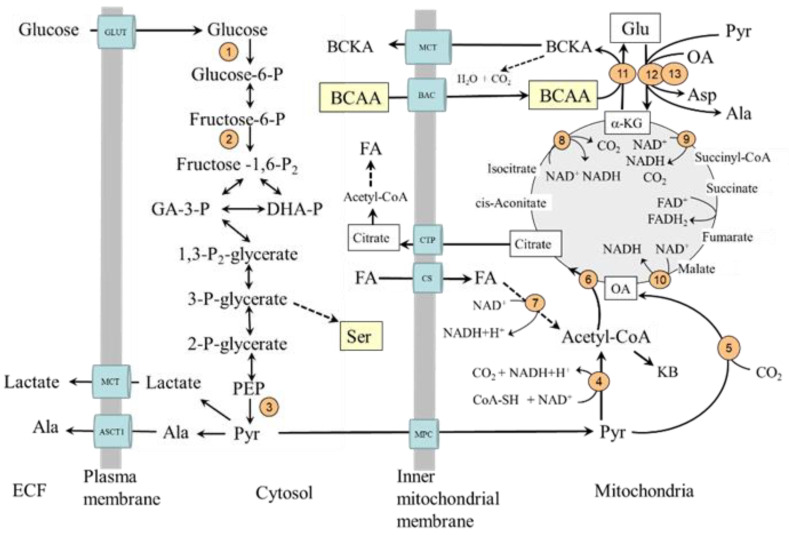
Glycolysis and its relationship to serine synthesis, beta-oxidation, citric acid cycle, and BCAA catabolism. 1, hexokinase; 2, phosphofructokinase; 3, pyruvate kinase; 4, pyruvate dehydrogenase; 5, pyruvate carboxylase; 6, citrate synthase; 7, beta-hydroxyacyl-CoA-dehydrogenase; 8, isocitrate dehydrogenase; 9, α-ketoglutarate dehydrogenase; 10, malate dehydrogenase; 11, BCAA aminotransferase; 12, AST; 13, ALT. BAC, branched-chain amino acid carrier (SLC25A44); BCAA, branched-chain amino acids; BCKA, branched-chain keto acids; CAC, citric acid cycle; CS, carnitine system; CTP citrate (tricarboxylate) transport protein; GLUT, glucose transporter; MCT, monocarboxylate transporter; MPC, mitochondrial pyruvate carrier; OA, oxaloacetate.

Glycolysis is the main pathway of the breakdown of glucose to pyruvate that occurs in the cytosol and provides the substrates for energy production as well as for storage of energy in the form of lipids (Figure 1). Insulin increases glucose disappearance from the blood and glycolysis by enhanced translocation of glucose from extracellular fluid to cytosol by activation of some glucose transporters (GLUT), primarily GLUT4, and of some glycolytic enzymes, specifically hexokinase, phosphofructokinase, and pyruvate kinase. The effects of insulin are determined by the type of tissue. For example, insulin increases the translocation of GLUT4 and hexokinase activity in muscles and adipocytes but not in the liver.

Pyruvate, the final product of glycolysis, can be in cytosol converted to alanine or lactate or transported from the cytosol to the mitochondria by one of two types of mitochondrial pyruvate carrier proteins. In mitochondria, pyruvate can be converted by pyruvate dehydrogenase (PDH) to acetyl coenzyme A (acetyl-CoA), the initial substrate for the CAC, by pyruvate carboxylase to oxaloacetate, and by alanine aminotransferase (ALT) to alanine.

The PDH activity is regulated by the phosphorylation/dephosphorylation of the enzyme. Its kinase is activated (i.e., the enzyme is inactivated) by increases in acetyl-CoA to CoA, ATP to ADP, and NADH to NAD^+^ ratios. Insulin activates PDH by reducing its phosphorylation and acetyl-CoA production from fatty acid oxidation. Pyruvate carboxylase is activated by acetyl-CoA, glucagon, and adrenaline and inhibited by insulin. Therefore, in the liver, its activation would promote gluconeogenesis by making more oxaloacetate be converted to phosphoenolpyruvate. In other tissues, primarily in the muscles, oxaloacetate is utilized in the CAC. The condensation reaction of oxaloacetate with acetyl-CoA to citric acid by citrate synthase is recognized as the rate-limiting step in the flux of the acetyl-CoA through the cycle regardless of whether the source of acetyl-CoA is glucose, fatty acids, or amino acids.

The CAC is the main source of reducing equivalents that enter the respiratory chain, where ATP is produced. The intermediates of the CAC play a role in the metabolism of several amino acids, such as glutamate, glutamine, aspartate, phenylalanine, tyrosine, tryptophan, threonine, and BCAA.

### Glycolysis and Fatty acid Oxidation in Diabetes

A common feature of both types of diabetes is impaired entry of glucose from extracellular space to the cell, decreased glycolysis, and mitochondrial dysfunction in most tissues [8,9,10,11,12,13]. In addition to the limited utilization of glucose, the utilization of fatty acids is of crucial importance [14]. The preferential fatty acid oxidation increases the mitochondrial ratios of acetyl-CoA to CoA and NADH to NAD^+^. The results are decreased acetyl-CoA synthesis from pyruvate and flux through the CAC, mainly due to the inhibition of NADH-producing enzymes, specifically malate dehydrogenase, isocitrate dehydrogenase, and α-ketoglutarate dehydrogenase and increased use of acetyl-CoA for the synthesis of ketone bodies (Figure 1). Hence, during diabetes, the flux through the CAC decreases [11,15]. It is very likely that these alterations have a fundamental role in impaired mitochondrial respiration and energy balance observed in the muscles, hearts, and kidneys of subjects with diabetes [4,16,17,18,19,20].

## 3. L-Serine and Diabetes

### 3.1. Basic Data on L-Serine Metabolism

**Figure 2:**
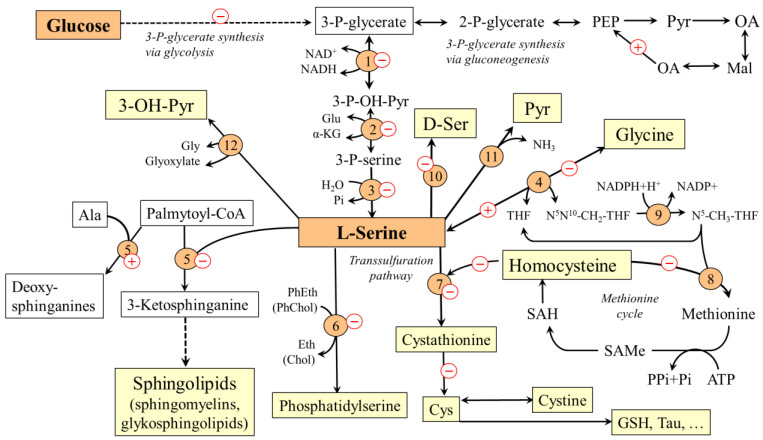
Main pathways of L-serine metabolism and their alterations during diabetes mellitus. The pluses and minuses indicate the predicted changes in diabetes. 1, 3-phosphoglycerate dehydrogenase; 2, phosphoserine aminotransferase; 3, phosphoserine phosphatase; 4, serine hydroxymethyltransferase; 5, serine palmitoyltransferase; 6, phosphatidylserine synthase; 7, cystathionine β-synthase; 8, methionine synthase; 9, methylene tetrahydrofolate reductase; 10, racemase 11, serine dehydratase; 12, serine-glyoxylate transaminase. Chol, choline; Eth, ethanolamine; GSH, glutathione; OA, oxaloacetate; Mal, malate; Pyr, pyruvate; PEP, phosphoenolpyruvate; PhChol, phosphatidylcholine; PhEth, phosphatidylethanolamine; SAH, S-adenosylhomocysteine; SAMe, S-adenosylmethionine; 3-OH-Pyr, 3-hydroxypyruvate.

It has been estimated that ~73% of L-serine appearance rate in fasting humans is the result of serine synthesis from 3-phosphoglycerate (3-PG), the intermediate in the glycolysis pathway, and from glycine [21]. The first step of L-serine synthesis from 3-PG is the oxidation of 3-PG to 3-phosphohydroxypyruvate, which is converted by 3-phosphoserine aminotransferase to 3-phosphoserine. The final step is the irreversible hydrolysis of 3-phosphoserine to L-serine by phosphoserine phosphatase (Figure 2). It is generally accepted that the biosynthetic flux of L-serine from 3-PG is controlled by the last step through feedback inhibition [22,23]. From glycine, L-serine can be synthesized by the enzyme serine hydroxymethyltransferase, which catalyzes the reversible conversions of glycine and 5,10-methylenetetrahydrofolate (N^5^N^10^-CH_2_-THF) to L-serine and tetrahydrofolate (THF). L-serine synthesis from 3-PG and glycine is high in many tissues, including the kidneys, brain (especially astrocytes), liver, and spleen [24,25]. L-serine synthesis in the liver is activated under conditions of increased glycolysis and decreased gluconeogenesis, such as consumption of a carbohydrate-rich diet [21,26,27].

L-serine is a substrate for the synthesis of proteins, phospholipids, particularly phosphatidylserine, and sphingolipids, such as ceramides, phosphosphingolipids, and glycosphingolipids, which are in large amounts in the white matter of the brain and in the myelin sheaths of nerves. L-serine acts as an agonist of the glycine receptor and, therefore, is classified as an inhibitory neurotransmitter [28,29]. L-serine, in reaction with homocysteine catalyzed by cystathionine β-synthase, initiates the transsulfuration pathway. This makes L-serine important for homocysteine disposal and synthesis of several sulfur-containing substances, such as cysteine, cystine, taurine, and glutathione. The connection of L-serine with folate and methionine cycles enables its role in the synthesis of nucleotides and many methylation reactions. Neurological abnormalities observed in primary disorders of its synthesis indicate that the amounts of L-serine provided by food may not always be sufficient and that L-serine should be classified as a conditionally essential amino acid [30].

### 3.2. Why L-Serine Levels Decrease in Diabetes

L-serine concentrations in plasma and tissues decrease in both T1DM [4,5,31,32] and T2DM [5,6,33,34,35,36,37]. The decrease in L-serine levels is probably due to two reasons. Firstly, due to decreased glycolysis and subsequent decrease in the supply of 3-P-glycerate, the L-serine synthesis decreases in most tissues. Secondly, L-serine may be deaminated by serine dehydratase to pyruvate or converted by serine-glyoxylate aminotransferase into hydroxypyruvate and, ultimately, glucose (Figure 2). Therefore, increased gluconeogenesis, which is one of the main metabolic features of diabetes, increases L-serine catabolism in the liver and the kidneys.

### 3.3. Consequences of L-Serine Deficiency in Diabetes

Due to the exceptional importance of L-serine in a broad range of metabolic reactions and cellular functions, the consequences of L-serine deficiency are numerous. Clinically important are disturbances in synthesis of sphingolipids, glycine deficiency, and hyperhomocysteinemia.

#### 3.3.1. Disturbances in Synthesis of Sphingolipids and Diabetic Neuropathy

A proven consequence of L-serine deficiency is impaired synthesis of sphingolipids, particularly ceramides and phospholipids [38,39,40,41]. Moreover, due to the possibility of substitution of L-serine by L-alanine during the first step of synthesis of sphingolipids by serine palmitoyl transferase, neurotoxic deoxysphinganines, which lack the C1 hydroxyl group of L-serine and therefore cannot be used for the synthesis of complex sphingolipids, are formed [34,42]. These substances accumulate in tissues and exert detrimental effects on neurite formation [39]. Hence, it is very likely that L-serine deficiency participates in the pathogenesis of diabetic neuropathy that may affect both limbs (peripheral type) and internal organs (autonomic type). Since deoxysphinganines are toxic to β-cells of the pancreas, their increased level may contribute to the pathogenesis of diabetes itself [41]. There are several studies reporting that L-serine supplementation reduces concentrations of deoxysphingolipids and manifestations of symptoms of diabetic neuropathy [5,40,43,44].

#### 3.3.2. Glycine Deficiency

Adaptive response to L-serine deficiency due to its impaired synthesis from 3-PG and increased catabolism in gluconeogenesis is its increased synthesis from glycine by L-serine hydroxymethyltransferase. The reaction requires N^5^N^10^-CH_2_-THF, that is formed during the degradation of glycine by a glycine cleavage system. Therefore, two molecules of glycine may be consumed during the synthesis of one molecule of L-serine:

Gly + NAD^+^ + THF → NH_3_ + CO_2_ + NADH + H^+^ + N^5^N^10^-CH_2_-THF (cleavage system)

Gly + N^5^N^10^-CH_2_-THF → L-Ser + THF (serine hydroxymethyltransferase)

Sum: 2 Gly + NAD^+^ → L-Ser + NADH + H^+^ + NH_3_ + CO_2_

Glycine levels decrease along with the decrease in L-serine levels in both types of diabetes [2,4,45,46,47]. However, it is not clear whether glycine deficiency in patients with diabetes affects some important physiological functions of glycine, such as neurotransmission, conjugation of bile acids, and synthesis of collagen, creatine, glutathione, heme, and purines. It is likely that an adaptive increase in L-serine synthesis from glycine plays a role in hyperhomocysteinemia and impaired synthesis of sulfur-containing substances (next item).

#### 3.3.3. Hyperhomocysteinemia and Impaired Synthesis of Sulfur-Containing Substances

L-serine deficiency can lead to an increase in homocysteine levels in two ways. The first is decreased supply of N^5^-CH_3_-THF for homocysteine methylation to methionine due to the adaptive increase in L-serine synthesis from glycine [48]. The second is an impaired synthesis of cystathionine from L-serine and homocysteine by cystathionine β-synthase and a subsequent decrease in the drain of homocysteine from the methionine cycle to the transsulfuration pathway (Figure 2). The possibility is supported by the presence of hyperhomocysteinemia in humans and rodents with cystathionine β-synthase deficiency [49].

Hyperhomocysteinemia is routinely observed in patients with diabetes and seems to be involved in an increased risk of cardiovascular, cerebrovascular, and thromboembolic diseases [50,51]. Decreased cytathionine synthesis due to L-serine deficiency may also be involved in impaired synthesis and alteration in several sulfur-containing substances, such as cysteine, cystine, taurine, and glutathione, reported in the serum of patients with diabetes [52]. Low levels of cysteine associated with increased homocysteine levels in diabetes have been reported by Rehman et al. [53].

Unfortunately, there are no studies on the effect of L-serine supplementation on levels of sulfur-containing substances in patients with diabetes. It has only been shown that L-serine administration decreases plasma homocysteine levels in hyperhomocysteinemia induced by high methionine diet [54,55,56].

## 4. The BCAA and *Diabetes*

### 4.1. Basic Data on BCAA Metabolism

**Figure 3:**
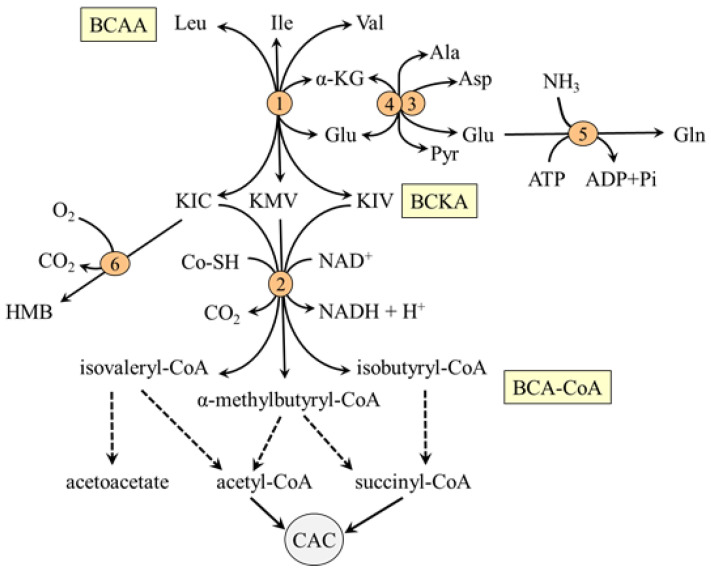
Main pathways of BCAA catabolism. 1, BCAA aminotransferase; 2, BCKA dehydrogenase; 3, AST; 4, ALT; 5, glutamine synthetase; 6, KIC dioxygenase. BCAA, branched-chain amino acids; BCA-CoA, branched-chain acyl-CoA; BCKA, branched-chain keto acids; CAC, citric acid cycle; HMB, β-hydroxy-β-methylbutyric acid; OA, oxaloacetate.

The BCAA are nutritionally essential amino acids that, together with their metabolites, the branched-chain keto acids (BCKA) and β-hydroxy-β-methylbutyric acid (HMB), are involved in the regulation of key protein-anabolic pathways and serve as an energy fuel during exercise and severe illness. Unlike most other amino acids, BCAA catabolism does not begin in the liver, but in extrahepatic tissues, especially in muscles. The cause is the negligible hepatic activity of BCAA aminotransferase, the first enzyme in a cascade of BCAA catabolism reactions (Figure 3), whereas its activity is high in muscles.

The BCAA aminotransferase enables the reversible transfer of amino group between BCAA and α-KG to form BCKA and glutamate (BCAA + α-KG ↔ BCKA + Glu). Glutamate produced in muscles by BCAA aminotransferase is used by mitochondrial alanine aminotransferase (ALT) and aspartate aminotransferase (AST) as a source of nitrogen for the synthesis of alanine (Glu + pyruvate → α-KG + Ala) and aspartate (Glu + oxaloacetate → α-KG + Asp), respectively. Since the BCAA aminotransferase reaction responds rapidly to changes in concentrations of its reactants, the removal of glutamate and the regeneration of α-KG by ALT and AST are essential for a continuous flux of the BCAA through the BCAA aminotransferase.

Alanine is transported from the mitochondria to the cytosol by an unknown carrier and is together with alanine synthesized in the cytosol released from muscles and used preferentially for glucose synthesis in the liver. Aspartate transported from the mitochondria to the cytosol by aspartate-glutamate carrier (AGC) is utilized in several reactions, such as the purine-nucleotide cycle and protein synthesis. Aspartate transamination back to oxaloacetate and its translocation back into the mitochondria via the malate-aspartate shuttle (specifically malate-ketoglutarate carrier) can be important for the continuous flux of the BCAA through the BCAA aminotransferase,

The second enzyme of BCAA catabolism is branched-chain α-keto acid dehydrogenase (BCKA dehydrogenase), which catalyzes irreversible decarboxylation of the BCKA to corresponding branched-chain acyl-CoA esters (BCA-CoA). At rest, the activity of BCKA in the muscles of a healthy individual is low. Therefore, most of the BCKA formed by BCAA aminotransferase is released from muscles and oxidized in tissues with high activity of BCKA dehydrogenase, such as the liver, heart, and kidneys, or aminated to the original BCAA. Increased concentrations of ATP, NADH, and acyl-CoA derivatives and decreased concentration of α-ketoisocaproate (KIC), the transamination product of leucine catabolism, inhibit the enzyme [57,58].

Beyond the BCKA dehydrogenase reaction, the metabolism of the BCAA diverges into separate pathways. The final products are acetoacetate, acetyl-CoA, and succinyl-CoA (Figure 3). It is estimated that 5–10% of KIC released to the blood is metabolized in the liver and kidneys by cytosolic enzyme KIC dioxygenase to produce HMB with favorable effects on protein balance and mitochondrial biogenesis in muscles [59].

### 4.2. Why the BCAA Increase in Diabetes

**Figure 4:**
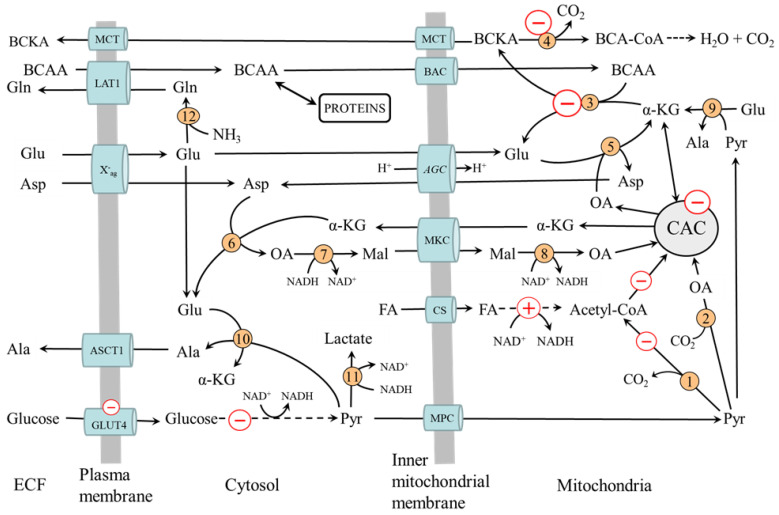
Muscle BCAA metabolism in diabetes. The pluses and minuses indicate the main changes associated with decreased glycolysis and preferential fatty acid oxidation resulting in impaired BCAA catabolism. 1, pyruvate dehydrogenase; 2, pyruvate carboxylase; 3, BCAA aminotransferase; 4, BCKA dehydrogenase; 5, AST mitochondrial; 6, AST cytosolic; 7, cytosolic malate dehydrogenase; 8, mitochondrial malate dehydrogenase 9, ALT mitochondrial; 10, ALT cytosolic; 11, lactate dehydrogenase; 12, glutamine synthetase. AGC, aspartate-glutamate carrier; ASCT1, alanine, serine, cysteine, and threonine carrier 1 (SLC1A4); BCAA, branched-chain amino acids; BAC, branched-chain amino acid carrier (SLC25A44); BCA-CoA, branched-chain acyl-CoA; BCKA, branched-chain keto acids; CAC, citric acid cycle; CS carnitine system; ECF, extracellular fluid; LAT1 (large neutral amino acid transporter 1); Mal, malate; MCT, monocarboxylate transporter; MKC, malate-ketoglutarate carrier; OA, oxaloacetate; MPC, mitochondrial pyruvate carrier; X^-^_ag_, a transporter for aspartate and glutamate (SLC1 family).

The possible causes of elevated BCAA levels in diabetes have been reviewed recently [60,61]. Supposed is impaired BCAA transamination and decarboxylation in muscles due to the changes associated with decreased glycolysis and preferential fatty acid oxidation (Figure 4). These are mainly:

- Decreased flux through the CAC, resulting in impaired α-KG supply to BCAA aminotransferase.
- Impaired conversion of glutamate to α-KG by AST and ALT in mitochondria due to decreased supply of oxaloacetate and pyruvate from glycolysis. The result is the drain of α-KG from the CAC (cataplerosis) and glutamate cumulation in mitochondria. A marked decrease in the rate of aspartate production from glutamate and oxaloacetate and a decrease in the Vmax of glutamate translocase was observed in heart mitochondria from the alloxan-diabetic rats compared to fed controls [62].
- Inhibition of BCKA dehydrogenase by increased levels of NADH and acyl-CoAs formed during β-oxidation.
- Increased BCAA release from the liver due to the activation of protein catabolism. The BCAA is released from the liver more than other amino acids because the activity of BCAA aminotransferase is very low in the liver.
- Increased transamination of BCKA to BCAA. It has been suggested that glutamine released from muscles can, under conditions of decreased activity of BCKA dehydrogenase, activate the synthesis of BCAA from BCKA or limit the transamination of BCAA to BCKA in visceral tissues [63,64].

The hypothesis of the role of impaired glycolysis in muscles in the pathogenesis of increased BCAA levels is supported by the blunted decline in plasma BCAA levels during the oral glucose tolerance test in subjects with insulin resistance or diabetes [65,66]. The fundamental importance of skeletal muscle is proven by high BCAA levels in muscles [4,67,68,69,70,71,72].

### 4.3. The Consequences of Increased BCAA Levels

#### 4.3.1. Insulin Resistance

There is a strong association of BCAA levels with insulin resistance, and the rise of BCAA in obesity is considered a prognostically significant factor in the development of T2DM [45,73,74]. The notion that elevations in BCAA levels contribute causally to insulin resistance is supported by the observation of impaired glucose disposal after BCAA infusion into circulation [75]. Several studies point to the role of the mTOR signaling pathway. It has been proposed that high levels of the BCAA increase via mTOR phosphorylation of insulin receptor substrate 1 (IRS-1), leading to the block of insulin signaling [76].

It should be emphasized that it is not quite sure that the effects of increased BCAA levels on mTOR signaling are detrimental in subjects with diabetes. The BCAA, particularly leucine, has potent anabolic effects and increases insulin release from pancreatic β-cells [77,78]. Therefore, under conditions of impaired insulin signaling, increased BCAA levels may promote anabolic reactions and prevent some negative consequences of insulin resistance or deficiency. Recent studies have shown that dietary supplementation with leucine attenuates insulin resistance, favors weight loss, and improves mitochondrial function [79,80,81]. Therefore, leucine supplementation is becoming a focus of attention in T2DM therapy.

#### 4.3.2. Accumulation of the BCAA Metabolites

It has been suggested that high BCAA levels interfere with fatty acid oxidation leading to the accumulation of acylcarnitines and acyl-CoAs with various lengths of carbon skeleton [82]. An increase in C3 and C5 acylcarnitines in animals fed by a high-fat diet supplemented with BCAA suggests that some of these acylcarnitines are the direct products of BCAA catabolism [45]. In recent years, attention has been given to the increased level of 3-hydroxyisobutyric acid, one of the valine metabolites [75,82,83,84]. The consequences of increased concentrations of the metabolites related to dysregulation of BCAA metabolism are not clear.

#### 4.3.3. The Increase in AAA Levels

The BCAA belongs together with aromatic amino acids (AAA; phenylalanine, tyrosine, and tryptophan) to a group of large neutral amino acids, which compete with each other for transport through plasma membranes by the same transporter referred to as the LAT1 (CLC7A5). Therefore, the rise of AAA is apparently caused by their reduced transport to the tissues due to the rise of the BCAA.

It has been suggested the elevation in the BCAA levels reduces the brain uptake of AAA, which are precursors of some neurotransmitters, notably dopamine and 5-hydroxytryptamine (serotonin), which may affect mood, cognition functions, hormone secretion (prolactin, cortisol), and the onset of fatigue [85]. Significant associations of the sum of the BCAA and AAA levels with insulin resistance and future diabetes have been reported [45,73,74,86].

#### 4.3.4. Alterations in Glutamate, Aspartate, Alanine, and Glutamine Levels

In the previous part of this article, it was shown that the BCAA metabolism is closely linked to the metabolism of glutamate, aspartate, alanine, and glutamine. However, the reports on changes in the levels of these amino acids are not consistent, and both increased and decreased plasma concentrations have been reported in subjects with diabetes [2,4,5,6,7]. Several speculations make it possible to explain these inconsistent findings. The decrease in glutamate synthesis due to the block in the flux of the BCAA through BCAA aminotransferase may cause a decrease in the concentrations of aspartate, alanine, and glutamine. The likely mechanism leading to increased levels of alanine in other subjects with diabetes may be the impaired entry of pyruvate to the CAC and its subsequent shift from pyruvate dehydrogenase to alanine aminotransferase and lactate dehydrogenase reactions. The suggestion is consistent with elevated lactate levels in patients with diabetes [47]. The cause of decreased alanine levels in other patients might be due to its increased consumption for gluconeogenesis in the liver.

## 5. Summary and Conclusions

The issue of diabetes is very complex, and in addition to genetic factors and obesity, other influences such as stress, alterations in the immune system, drugs, nutritional habits, physical activity, and changes in gut microbiota are also involved in the etiopathogenesis of diabetes and may affect amino acid metabolism. The focus of this article is specifically the changes in amino acid metabolism due to impaired glycolysis.

In the article is demonstrated that decreased L-serine and increased BCAA levels in subjects with diabetes are directly related to impaired glycolysis, preferential use of fatty acids as an energy substrate, and decreased flux through the CAC and that these alterations are implicated in the development of several complications. L-serine deficiency contributes to the altered synthesis of sphingolipids, which plays a role in the pathogenesis of diabetic neuropathy, hyperhomocysteinemia due to impaired homocysteine disposal via the methionine cycle and transsulfuration pathway, and glycine deficiency due to the adaptive increase in glycine utilization for L-serine synthesis. Enhanced BCAA levels contribute to increased levels of aromatic amino acids (phenylalanine, tyrosine, and tryptophan), insulin resistance, and accumulation of various metabolites whose influence on the progression of diabetes has not been clarified. Due to the positive effects of BCAA on protein balance, it is not clear whether their increased levels in diabetes should be recognized as beneficial or harmful. It is concluded that:

(i) Plasma amino acid concentrations should be monitored in patients with diabetes, and systematic investigation is needed to examine the effects of L-serine and glycine supplementation on diabetes progression in the case of a decrease in the level of these amino acids in the blood.
(ii) The ratio between BCAA and L-serine levels could be a better prognostic indicator of insulin deficiency or resistance than BCAA alone.
(iii) A better understanding of the consequences of perturbations in BCAA metabolism is essential for making decisions regarding dietary recommendations in patients with diabetes.

## Data Availability

Not applicable.
